# Glyphosate and AMPA levels in human urine samples and their correlation with food consumption: results of the cross-sectional KarMeN study in Germany

**DOI:** 10.1007/s00204-020-02704-7

**Published:** 2020-03-30

**Authors:** Sebastian T. Soukup, Benedikt Merz, Achim Bub, Ingrid Hoffmann, Bernhard Watzl, Pablo Steinberg, Sabine E. Kulling

**Affiliations:** 1 Department of Safety and Quality of Fruit and Vegetables, Max Rubner-Institut, Haid-und-Neu-Straße 9, 76131 Karlsruhe, Germany; 2 Department of Physiology and Biochemistry of Nutrition, Max Rubner-Institut, Karlsruhe, Germany; 3 Department of Nutritional Behaviour, Max Rubner-Institut, Karlsruhe, Germany; 4 Max Rubner-Institut, Karlsruhe, Germany

**Keywords:** Glyphosate, Biomonitoring, Urine, Food consumption, Correlation, Human

## Abstract

Glyphosate (*N*-[phosphonomethyl]-glycine) is the most widely used herbicide worldwide. Due to health concerns about glyphosate exposure, its continued use is controversially discussed. Biomonitoring is an important tool in safety evaluation and this study aimed to determine exposure to glyphosate and its metabolite AMPA, in association with food consumption data, in participants of the cross-sectional KarMeN study (Germany). Glyphosate and AMPA levels were measured in 24-h urine samples from study participants (*n* = 301). For safety evaluation, the intake of glyphosate and AMPA was calculated based on urinary concentrations and checked against the EU acceptable daily intake (ADI) value for glyphosate. Urinary excretion of glyphosate and/or AMPA was correlated with food consumption data. 8.3% of the participants (*n* = 25) exhibited quantifiable concentrations (> 0.2 µg/L) of glyphosate and/or AMPA in their urine. In 66.5% of the samples, neither glyphosate (< 0.05 µg/L) nor AMPA (< 0.09 µg/L) was detected. The remaining subjects (*n* = 76) showed traces of glyphosate and/or AMPA. The calculated glyphosate and/or AMPA intake was far below the ADI of glyphosate. Significant, positive associations between urinary glyphosate excretion and consumption of pulses, or urinary AMPA excretion and mushroom intake were observed. Despite the widespread use of glyphosate, the exposure of the KarMeN population to glyphosate and AMPA was found to be very low. Based on the current risk assessment of glyphosate by EFSA, such exposure levels are not expected to pose any risk to human health. The detected associations with consuming certain foods are in line with reports on glyphosate and AMPA residues in food.

**Electronic supplementary material:**

The online version of this article (10.1007/s00204-020-02704-7) contains supplementary material, which is available to authorized users.

## Introduction

**Fig. 1:**
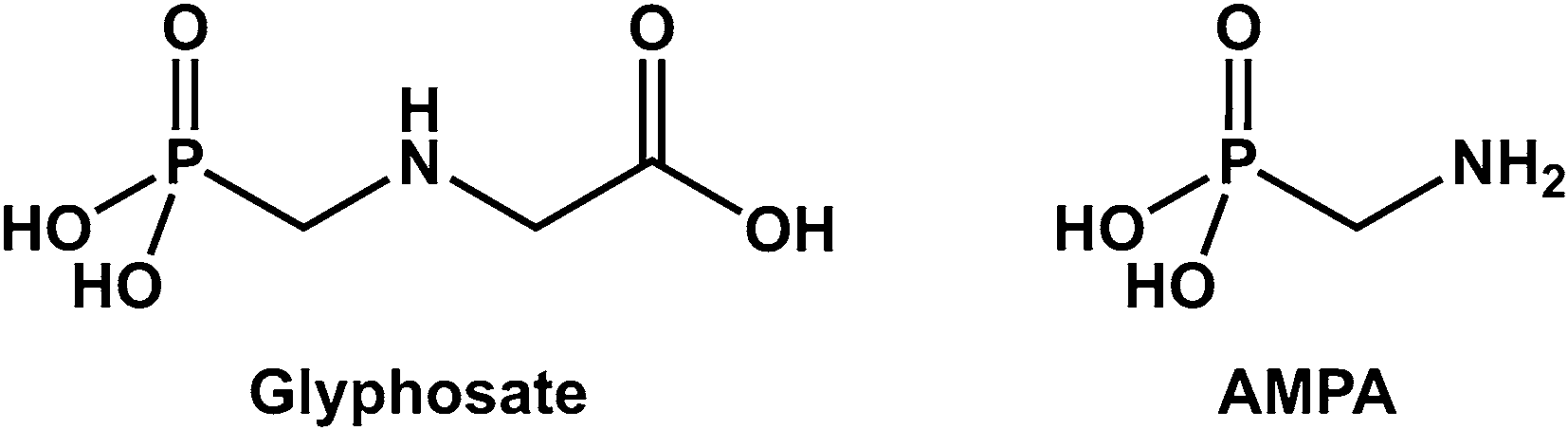
Chemical structures of glyphosate (*N*-[phosphonomethyl]-glycine) and aminomethylphosphonic acid (AMPA)

Glyphosate (*N*-[phosphonomethyl]-glycine; Fig. 1) is a broad-spectrum herbicide most frequently used as the active ingredient in agricultural formulations worldwide. In 2014, the global use of glyphosate-based formulations was approximately 825,000 tons applied to a total area of about 400 million hectares worldwide (Benbrook 2016). Glyphosate is widely used in agriculture for various purposes, not only weed control but also reduced soil tillage, seed bed preparation, stubble management, and pre-harvest application (desiccation). Pre-harvest treatment is used for cereals, rapeseed, and pulses to reduce weed density as well as grain moisture content to achieve faster and more uniform maturation.

Glyphosate inhibits 5-enolpyruvylshikimate 3-phosphate synthase, a key enzyme in the biosynthetic pathway of essential aromatic amino acids, which consequently strongly affects protein biosynthesis in plants and limits their growth. Aminomethylphosphonic acid (AMPA; Fig. 1) is the main metabolite of glyphosate in the environment and is formed by microbial degradation in the soil (Franz et al. 1997). In the US, an actual chronic population adjusted dose (cPAD) of 1.0 mg/kg body weight (BW)/day for glyphosate was established by the Environmental Protection Agency (EPA), derived from a maternal no-observed-adverse-effect level (NOAEL) of 100 mg/kg BW/day from a pre-natal developmental toxicity study in rabbits and by applying an uncertainty factor (UF) of 100 (EPA 2018). In the EU, the acceptable daily intake (ADI) of glyphosate is 0.5 mg/kg BW/day, based on the maternal and developmental NOAEL of 50 mg/kg BW/day from a developmental toxicity study in rabbits and by applying a standard UF of 100 (EFSA 2015). The acute reference dose (ARfD) is 0.5 mg/kg BW, based on the NOAEL of 50 mg/kg BW/day from a developmental toxicity study in rabbits, due to the occurrence of severe toxicity including mortality observed in pregnant rabbits and the increased incidence of post-implantation losses observed in two out of seven developmental toxicity studies in rabbits, and by applying an UF of 100 (EFSA 2015).

Humans may be exposed to glyphosate in occupational as well as environmental settings (OCSPP, Office of Chemical Safety and Pollution Prevention 2017) through various routes such as food and drinking water. Foods with potential higher glyphosate residue levels include some dried pulses and some cereals (EFSA 2018). In the EU, this is in part reflected by higher glyphosate maximum residue levels (MRLs), such as for oats (20 mg/kg), barley (20 mg/kg), wheat (10 mg/kg), lentils (10 mg/kg), beans (2 mg/kg), peas (10 mg/kg), and canola seeds (10 mg/kg) (EU, European Union 2013).

Valid data on the extent of human exposure to glyphosate in the general population are essential for scientifically substantiated risk assessments. Human exposure can be monitored by measuring the urinary levels of glyphosate and AMPA, although the available data sets are very limited in number (Gillezeau et al. 2019). EFSA reported that published glyphosate levels in human urine samples resulting from dietary intake of glyphosate represented 0.1–0.66% of the ADI, while the maximum levels of AMPA in human urine samples were estimated to remain below 0.1% of the ADI (EFSA 2015). Interestingly, there was no direct correlation between glyphosate and AMPA (EFSA 2015).

Regarding human exposure to glyphosate in Germany, only one report in a peer-reviewed journal could be identified. The German Environment Agency (Umweltbundesamt) monitored glyphosate and AMPA levels in 24-h urine samples from young adults between 2001 and 2015 with a GC-MS/MS technique (Conrad et al. 2017). The urinary concentrations of glyphosate and AMPA were at or above the limit of quantitation (0.1 µg/L in both cases) in about 32 and 40% of the 399 samples analyzed (15 samples each year), respectively. The maximum values were 2.80 µg glyphosate/L and 1.88 µg AMPA/L, both of them measured in samples from 2013. With regard to studies from additional countries dealing with human exposure to glyphosate, we refer to the current review of Gillezeau et al. (2019). This review listed three studies investigating the association between glyphosate/AMPA exposure and food consumption or dietary pattern in the general population (no occupational exposure) (Knudsen et al. 2017; McGuire et al. 2016; Parvez et al. 2018). The number of participants in these three studies was between 27 and 71, and one of the studies (McGuire et al. 2016) only examined the differences in urinary glyphosate and AMPA concentrations between subjects consuming organic and conventionally grown food. Thus, urinary glyphosate/AMPA measurements in a well-characterized study population with available data regarding food consumption are lacking.

To fill this gap, we performed further analyses in biological samples from volunteers who participated in the cross-sectional Karlsruhe Metabolomics and Nutrition (KarMeN) study. The KarMeN study was conducted at the Max Rubner-Institut in Karlsruhe, Germany. The main aim was to characterize the blood plasma and urine metabolome of healthy women and men (range 18–80 years) by targeted and non-targeted metabolite profiling, and to assess the influence of sex, age, body composition, diet, and physical activity on the metabolite profiles of the participants (Armbruster et al. 2018; Biniaminov et al. 2018; Bub et al. 2016; Krüger et al. 2017; Mack et al. 2018; Merz et al. 2018; Rist et al. 2017).

The main objective was to determine the glyphosate exposure of a metabolically very well-characterized population of around 300 participants in association with food consumption data collected via a 24-h dietary recall.

## Materials and methods

### Study design

The KarMeN Study was a cross-sectional study conducted at the Max Rubner-Institut in Karlsruhe, Germany, between 2012 and 2013, aiming to determine the impact of a number of factors on the human metabolome in healthy men and women. Study design and examination procedures were described in detail elsewhere (Bub et al. 2016). In brief, a total of 312 volunteers aged 18–80 years were recruited. Exclusion criteria were smoking, acute or regular medication including hormonal contraceptives for women, illness requiring treatment, supplement use and, additionally, pregnancy or breast-feeding. Each individual visited the study center three times for detailed characterization (Bub et al. 2016). The study was registered at the German Clinical Trials Register (No. DRKS00004890) and conducted after its approval by the local ethics committee (State Medical Chamber of Baden-Württemberg, Stuttgart, Germany, F-2011-051), according to the guidelines of the Declaration of Helsinki. All participants gave written informed consent prior to participating in the study.

### 24-h urine collection

Participants were examined by trained study personnel according to standard operating procedures, and anthropometric, clinical, and functional parameters were assessed (Bub et al. 2016). Participants collected their urine for 24 h on the day prior to their examination including the first morning urine. Collection bottles were stored in a refrigerator or in cool bags with cooling pads for transportation. Upon delivery of the collected 24-h urine samples to the study center, the volume was recorded, 2 × 14 mL were centrifuged at 1850×*g* at 20 °C and aliquoted into small portions. All samples were initially frozen at −20 °C for 1 day and then cryopreserved in liquid nitrogen at −196 °C until analysis.

### Glyphosate and AMPA measurements in 24-h urine samples

Glyphosate and AMPA were quantified in the 24-h urine samples by LC-MS/MS according to the method of Jensen et al. (2016) with the following minor modifications. ^13^C_2_,^15^*N*-glyphosate (precursor/product ion transitions: 171/63 and 171/126) instead of ^13^C_3_,^15^*N*-glyphosate was used as the internal standard. Matrix-matched calibration samples instead of calibration standards in pure solvent were prepared for quantitation. For this purpose, a mixture of two analyte-free urine samples was used. To reach the final target concentration of 0.1% formic acid (v/v) in all samples at the end of the sample preparation, the working calibration standard solutions (glyphosate and AMPA) as well as the working internal standard solution (D_2_,^13^C,^15^N-AMPA and ^13^C_2_,^15^*N*-glyphosate) were prepared in 1.3% (v/v) formic acid in water.

Glyphosate and D_2_,^13^C,^15^N-AMPA (both purchased from Sigma-Aldrich Chemie GmbH, Taufkirchen, Germany) as well as AMPA and ^13^C_2_,^15^*N*-glyphosate (both purchased from LGC Standards GmbH, Wesel, Germany) were obtained as aqueous solutions (certified reference material). The LC-MS/MS analyses were performed on a QTrap 5500 mass spectrometer (AB Sciex, Darmstadt, Germany) equipped with a Nexera LC system (Shimadzu, Duisburg, Germany).

After establishing the method, verification of the validation parameters (accuracy, precision, matrix effect, linearity, limit of detection [LOD], limit of quantitation [LOQ], and selectivity) was performed. The results are summarized in the Supplemental Material: verification of validation parameters.

### Dietary assessment

Trained study personnel assessed the food consumption of each individual (in g/day) on two non-consecutive days at least 2 weeks apart in a personal and telephone interview using 24-h dietary recalls with the validated software EPIC-Soft (Slimani et al. 1999, 2000). The first 24-h recall covered the same period as the 24-h urine sample collection. Participants used standard units (e.g. a slice of bread), household measurements (e.g. a tablespoon), and a picture booklet providing photographs of portion sizes for various foods to indicate the consumed amount per meal.

Based on the national reports on residues of plant protection products between 2011 and 2017 from the Federal Office of Consumer Protection and Food Safety (BVL) in Germany, we identified foods that were reported to be considerably contaminated with glyphosate and/or AMPA, including pulses (dried lentils, dried peas, and dried beans), cereals (wheat, rye, and buckwheat), soy, mushrooms, and honey (BVL 2013, 2014, 2015, 2016, 2017, 2018, 2019). The data basis used for this evaluation is based on the analysis results submitted to the BVL from the Federal States (Länder). Since a number of German beers were reported to contain significant levels of glyphosate (Munich Environmental Institute 2016, 2017), beer was also included in the further analyses. These foods, except cereals, which were grouped under the term “bread”, were then aggregated into food groups for further analyses (Supplementary Table 1).

### Statistical analyses

The values of the urinary concentrations of glyphosate and AMPA below LOD were set to zero. Values between LOD and LOQ (defined as traces) were considered as determined by a measurement; since they were lower than LOQ, these values are associated with a higher uncertainty. However, the advantage of using these real values instead of possibly replacing them by some imputation technique is that the ranks of the values are retained. This is important in our case, since Spearman rank correlation analyses were also used (see below).

To calculate the 24-h urinary excretion of glyphosate and AMPA, the measured urine concentrations were multiplied by the corresponding recorded 24-h urine volume of each participant.

Spearman correlation analyses were used to investigate the association between current (past 24 h) consumption of food groups and the concurrent 24-h urinary excretion of glyphosate, AMPA, or their sum. The Benjamini–Hochberg method was applied to adjust for multiple testing (Benjamini and Hochberg 1995). The Mann–Whitney-*U* test was used to test differences between consumers (> 0 g/day reported consumption) and non-consumers (0 g/day reported consumption) regarding the food groups of interest. Sensitivity analyses including covariate age and sex in partial Spearman correlation analyses were performed to investigate the effect of these covariates on the observed associations. Since the glomerular filtration rate was not associated with the excretion of glyphosate and AMPA metabolites in urine (data not shown), this variable was not included. All statistical analyses were performed using software SAS Version 9.4 (SAS Institute, Cary, NC, USA) with *p* values < 0.05 considered statistically significant.

### Toxicological safety evaluation

To estimate extent by which the current ADI value for glyphosate (0.5 mg/kg BW/day) in the European Union (EFSA 2015) is exhausted in our study population, the intake of glyphosate was calculated by assuming the following: about 20% of the ingested glyphosate is absorbed, glyphosate absorption occurs rapidly, the absorbed glyphosate is poorly metabolized and rapidly eliminated via urine, showing no potential for bioaccumulation (EFSA 2015). Hence, the 24-h urinary excretion data of glyphosate and AMPA were multiplied by 5 and divided by the corresponding body weight to calculate the intake of glyphosate per kg BW for each study participant. EFSA (2015) reported that AMPA shows a toxicological profile similar to glyphosate, thus additional calculations were also performed in a similar way for AMPA, as well as for the sum of glyphosate and AMPA.

## Results

### Study population

We excluded 11 individuals due to acute medication or illness requiring treatment. The study population analyzed included 301 individuals, 172 men (57.1%), and 129 women (42.9%) with a mean age of 44.4 and 51.7 years, respectively. The anthropometric data of the study population are shown in Table 1. Quantitative information about consumption of the food groups of interest is given in Supplementary Table 2.

**Table 1:** Anthropometric data and 24-h urinary excretion rates of glyphosate and AMPA

Variable^b^	Subgroup 1 (*n* = 200)	Subgroup 2 (*n* = 76)	Subgroup 3 (*n* = 25)	*p*_linear trend_^c^
Percentage of study population (%)	66.5	25.2	8.3	
Age (years)	49.0 ± 16.0	46.2 ± 19.0	39.6 ± 17.0	0.0088
Body mass index (kg/m^2^)	23.7 ± 2.7	24.3 ± 3.2	23.9 ± 2.9	0.8124
Men in each subgroup (%)	50.5	69.7	72.0	0.0046
Glyphosate (µg/24 h)	n.d.^d^	0.206 ± 0.142	0.446 ± 0.269	< 0.0001
Maximum	n.d	0.759	1.088	
Minimum	n.d	n.d	n.d	
AMPA (µg/24 h)	n.d	0.063 ± 0.127	0.202 ± 0.281	< 0.0001
Maximum	n.d	0.489	1.012	
Minimum	n.d	n.d	n.d	

The KarMeN study population (women + men) was divided into three subgroups, depending on whether glyphosate and/or AMPA was detected in the collected urine samples^a^

^a^Subgroup 1: neither glyphosate nor AMPA were detected. Subgroup 2: traces of glyphosate and/or AMPA were detected. Subgroup 3: glyphosate and/or AMPA levels were above the limit of quantitation

^b^Values are given as arithmetic mean ± standard deviation or percentage

^c^Chi-square test was used for categorical data, linear regression models were used to test a linear trend across subgroups

^d^*n.d*. not detected

### Glyphosate and AMPA exposure

**Fig. 2:**
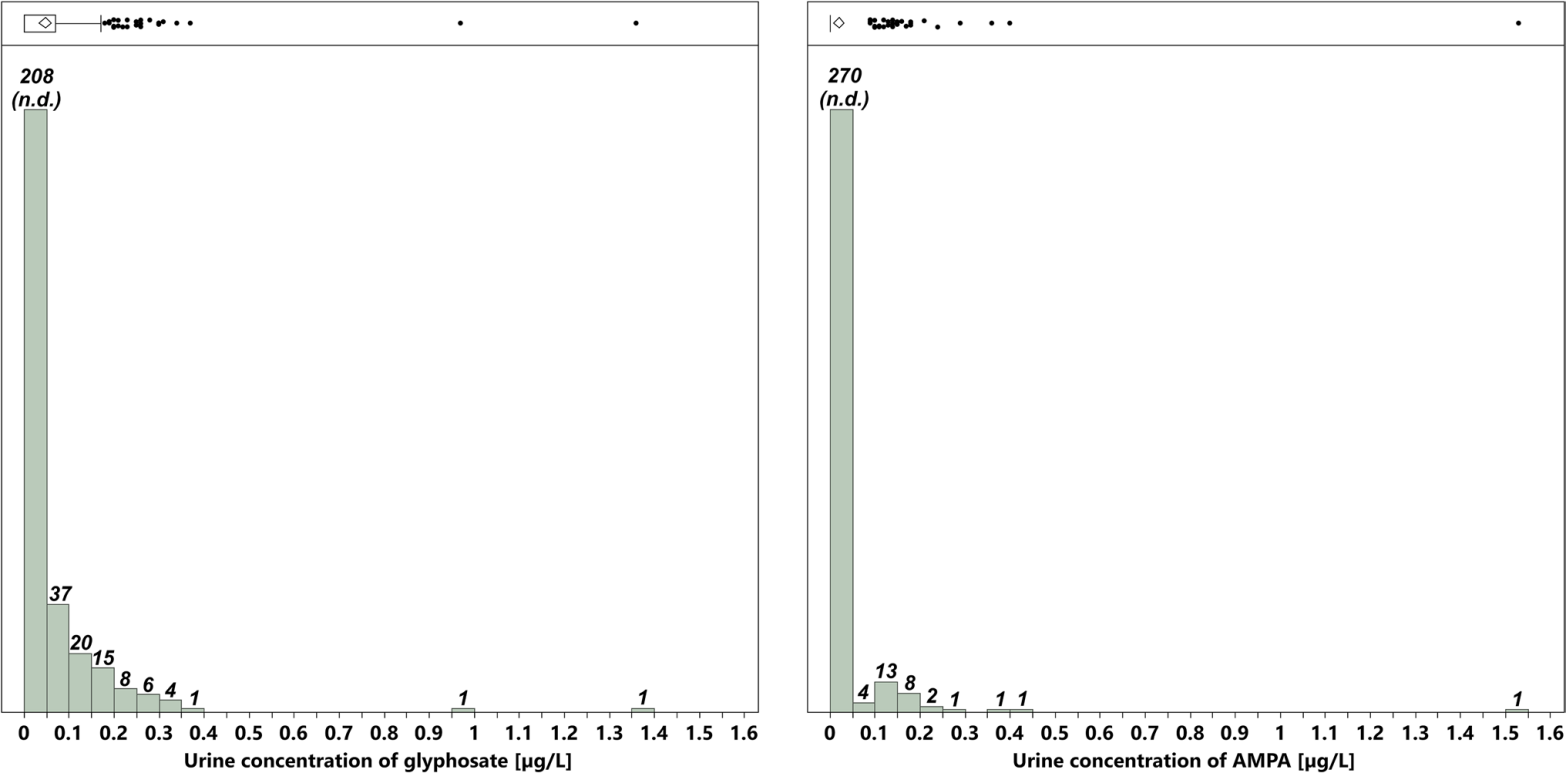
Distribution of the measured glyphosate and AMPA urine concentrations in the study population. The numbers displayed at the top of the columns are the number of samples per column

To determine the glyphosate and AMPA exposure in the study population, urine samples collected over 24 h were analyzed by a validated LC–MS/MS method. In 66.5% of the study population samples, neither glyphosate (LOD = 0.05 µg/L) nor AMPA (LOD = 0.09 µg/L) was detected (from now on called subgroup 1). Traces (values between LOD and LOQ) of glyphosate and/or AMPA were detected in 25.2% of the participants (subgroup 2). Only 8.3% of the subjects exhibited glyphosate and/or AMPA levels above the LOQ (LOQ = 0.2 µg/L for glyphosate and AMPA), the maximum concentrations measured being 1.36 and 1.53 µg/L, respectively (subgroup 3). The distribution and descriptive statistics of the measured glyphosate and AMPA urine concentrations revealed that participants with detectable levels of glyphosate (≥ 0.05 µg/L, *n* = 93) exhibited glyphosate urine concentrations with a median of 0.11 µg/L, an arithmetic mean of 0.16 µg/L and a range from 0.05 to 1.36 µg/L. The participants with detectable levels of AMPA (≥ 0.09 µg/L, *n* = 31) showed urine concentrations with a median of 0.14 µg/L, an arithmetic mean of 0.20 µg/L, and a range from 0.09 to 1.53 µg/L (Fig. 2; Table 2).

**Table 2:** Descriptive statistics of the measured glyphosate and AMPA urine concentrations

Analyte	Number of considered participants	Median (µg/L urine)	Arithmetic mean (µg/L urine)	Range (µg/L urine)
Glyphosate	93	0.11	0.16	0.05 – 1.36
AMPA	31	0.14	0.20	0.09 – 1.53

Only the participants with detectable levels of glyphosate (≥ 0.05 µg/L urine) or AMPA (≥ 0.09 µg/L urine) were taken into account. For the calculations, the values for traces (values between LOD and LOQ) were used as determined in the measurement instead of replacing them by imputation techniques (for more information see “Statistical analyses”)

The mean glyphosate and AMPA 24-h urinary excretion rates were 0.103 ± 0.175 and 0.052 ± 0.149 µg/24 h for men in subgroups 2 and 3, respectively, and 0.070 ± 0.172 and 0.007 ± 0.035 µg/24 h for women in subgroups 2 and 3, respectively (Table 1). A weak positive correlation (rho = 0.32, *p* < 0.0001, Supplementary Fig. 1) between the excreted amounts of glyphosate and the excreted amounts of AMPA was observed.

### Toxicological safety evaluation

**Fig. 3:**
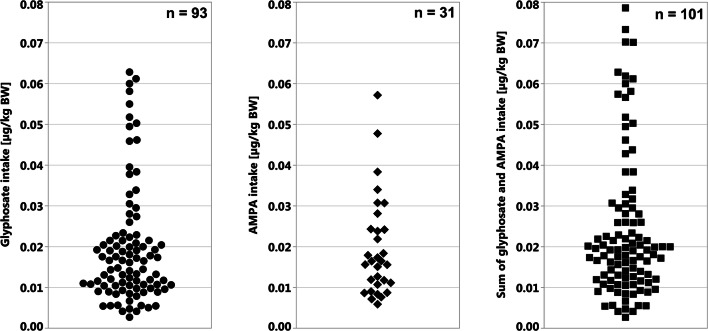
Calculated intakes of glyphosate (circle), AMPA (diamond), and the sum of both (square) in the study population. In each diagram, only the participants with detectable levels are displayed

Intakes of glyphosate, AMPA, and the sum of both were calculated to provide potential data about toxicology. In the case of glyphosate, the maximum intake level in our study population was 0.063 µg/kg BW, which corresponds to a 0.13‰ exhaustion of the ADI value. Another participant showed a maximum intake of AMPA of 0.057 µg/kg BW (0.11‰ exhaustion of the ADI value) and also exhibited the maximum intake of the sum of glyphosate and AMPA (0.079 µg/kg BW; 0.16‰ exhaustion of the ADI value) (Fig. 3).

### Associations between urinary glyphosate and/or AMPA excretion and concurrent food consumption

A positive Spearman correlation between the consumption of pulses and urinary glyphosate excretion (rho = 0.26, *p* < 0.0001) and between the consumption of mushrooms and urinary AMPA excretion (rho = 0.18, *p* = 0.0102) was observed. Furthermore, the sum of glyphosate and AMPA excretion was significantly associated with the consumption of pulses in our study population (rho = 0.24, *p* = 0.0003) (Table 3). The observed results were independent of age and sex, since adjustments for age and sex did not substantially alter the observed correlation coefficients (Supplementary Table 3).

**Table 3:** Spearman correlation coefficients and corresponding *p* values of associations between 24-h urine metabolite excretion and consumption of specific food groups

Food group (g/day)	Glyphosate (µg/24 h)		AMPA (µg/24 h)		Sum glyphosate + AMPA (µg/24 h)	
	rho	*p*	rho	*p*	rho	*p*
Honey	0.09	0.4758	− 0.05	0.7769	0.05	0.7769
Pulses	**0.26**	** < 0.0001**	− 0.01	0.9188	**0.24**	**0.0003**
Mushrooms	− 0.06	0.7769	**0.18**	**0.0102**	0.01	0.9188
Bread	0.05	0.7898	0.03	0.9013	0.03	0.3013
Beer	− 0.08	0.6866	0.03	0.9013	− 0.04	0.8880
Soy products	0.01	0.9188	0.02	0.9188	− 0.002	0.9725

Significant correlations are marked in bold; reported *p* values are corrected for multiple testing

Comparing consumers and non-consumers of selected food groups also revealed a significant correlation between the consumption of pulses and glyphosate excretion as well as between the consumption of mushrooms and AMPA excretion (Table 4, Supplementary Table 4; Supplementary Figs. 2–4).

**Table 4:** Mann–Whitney *U* test statistics (*p* values) for comparing 24-h urinary glyphosate and AMPA excretion rates between consumers and non-consumers of selected food groups

Food group (g/day)	Glyphosate (µg/24 h)	AMPA (µg/24 h)	Sum glyphosate + AMPA (µg/24 h)
Honey	0.0998	0.3106	0.3400
Pulses	** < 0.0001**	0.8852	** < 0.0001**
Mushrooms	0.3379	**0.0016**	0.8342
Bread	0.6278	0.9314	0.5012
Beer	0.1227	0.5525	0.3784
Soy products	0.8286	0.7515	0.9660

Significant differences are marked in bold

## Discussion

Glyphosate and/or AMPA were detected in around 1/3 of the KarMeN study population, with urine concentrations of up to 1.36 µg/L for glyphosate and 1.53 µg/L for AMPA: median and arithmetic mean glyphosate urine levels were 0.11 µg/L and 0.16 µg/L (only for participants with ≥ 0.05 µg/L, *n* = 93), and median and arithmetic mean AMPA urine levels were 0.14 µg/L and 0.20 µg/L (only for the participants with ≥ 0.09 µg/L, *n* = 31). In general, these results support outcomes from two previous studies with fewer participants in Europe. In one of those study in Germany in 2013, the same year as the KarMeN study, the maximum glyphosate and AMPA concentrations in 24-h urine samples (*n* = 39) were 2.80 µg/L (median: 0.11 µg/L) and 1.88 µg/L (median: < LOQ) (Conrad et al. 2017). More recently, Connolly et al. (2018) reported glyphosate levels of up to 1.35 µg/L (median: 0.87 µg/L, based on 10 out of 50 samples with > 0.5 µg/L) in spot urine samples from 50 Irish adults. By comparison, exposure to glyphosate seems to be somewhat higher in the US, where glyphosate concentrations of up to 7.20 µg/L (median 3.25 µg/L, mean 3.40 µg/L, *n* = 71) were recently reported in spot urine samples from pregnant women (Parvez et al. 2018). In conclusion, the measured urinary concentrations of glyphosate and AMPA in our study were in the lower range, and the estimated glyphosate and AMPA intake levels were clearly below the ADI value valid in the EU.

The second task of our study was to unravel possible associations between glyphosate and AMPA exposure levels and food consumption. Among the various food groups tested, significant associations were found for pulses and mushrooms. The significant correlation detected between the consumption of pulses and the excretion of glyphosate (*p* < 0.0001), as well as that of the sum of glyphosate and AMPA (*p* = 0.0003) may be a consequence of glyphosate being used as a pre-harvest desiccant (Benbrook 2016), which is allowed in various countries such as Canada, and also in the EU. Here it is applied to reduce the moisture content of harvested seeds and for perennial weed control. Glyphosate is administered only a few days before harvest, which may result in higher glyphosate and AMPA residues. Studies that measured glyphosate residues in diverse food items show that pulses and their products are always in the group with the highest glyphosate load (BVL 2013, 2014, 2015, 2016, 2017, 2018, 2019; Stephenson et al. 2018; Zoller et al. 2018). However, in almost all cases, the measured residues were below the legally tolerated maximum residue levels (MRLs), which in the case of lentils (dry) was raised from 0.1 mg/kg to 10 mg/kg in 2012 in the EU (EFSA 2012; European Union (EU) 2012).

The mean consumption of pulses in KarMeN participants in subgroup 3 was 18.8 g/day. Since most individuals in Western countries do not consume pulses every day, exposure to glyphosate by habitual consumption is expected to be much lower. Yet, dietary recommendations worldwide recommend increasing the consumption of pulses due to their health benefits and environmental sustainability (FAO 2016). Pulse consumption may thus increase and in turn lead to higher glyphosate exposure levels, although even then critical exposure levels may not be reached. Although the correlation of glyphosate excretion with the consumption of pulses is interesting and explainable, the findings should be interpreted with care, since it is only based on a low number of consumers with an appreciable pulse consumption within the KarMeN study.

The consumption of mushrooms correlated significantly with urinary AMPA excretion rates, but not with urinary glyphosate excretion rates, which is in line with National Reports on Plant Protection Product Residues of the Federal Office of Consumer Protection and Food Safety (BVL) in Germany (BVL 2013, 2014, 2015, 2016, 2017, 2018, 2019). These reports stated that AMPA, but not glyphosate, was almost exclusively detected in contaminated mushrooms. The majority of mushrooms consumed are button mushrooms, most likely cultivated button mushrooms, which are grown on a substrate commonly consisting of cereal straw in combination with dung. Cereals have been reported to be contaminated with glyphosate (BVL 2013, 2014, 2015, 2016, 2017, 2018, 2019; Stephenson and Harris 2016; Stephenson et al. 2018); thus, the resulting straw of contaminated cereals may contain significant amounts of glyphosate residues. A (microbial) degradation of glyphosate to AMPA in this substrate (Sviridov et al. 2015) with subsequent absorption of AMPA by the cultivated mushrooms could be a potential pathway leading to the observed associations.

No statistically significant associations between the excretion rates of either glyphosate or AMPA and the consumption of beer or bread could be observed in the KarMeN study. Unlike pulses and mushrooms, where potentially contaminated foods are consumed directly, bread and beer are products based on potentially contaminated cereals, which in turn may result in reduced contamination of the consumed foods. Furthermore, it is possible that the glyphosate concentrations in the 24-h urine samples were too low to be detected, considering the reported glyphosate concentrations for beer (Munich Environmental Institute 2016, 2017) in combination with the consumed quantity (mean consumption of 489 mL/day in the case of beer consumers) and the postulated absorption rate of 20% for glyphosate (EFSA 2015).

The same explanation might be true for honey and soy product consumption, where no significant association could be observed. While potentially contaminated honey is commonly consumed in small amounts (mean consumption of honey by consumers in the KarMeN study = 13.8 g/day), the resulting dietary glyphosate exposure is rather low. Furthermore, soy is rarely consumed directly and mostly as processed soy products such as tofu or soy drinks. Thus, a dilution effect might occur in the case of soy as postulated for beer and bread.

Our study has several strengths. First, we determined glyphosate exposure in a large number of very well-characterized participants. Second, rather than using spot urine samples, we collected 24-h urine samples and thus monitored the complete excretion of both glyphosate and AMPA using a validated analytical LC–MS method with isotopically labelled standards. Third, urine collection could be related to a concurrent 24-h dietary recall.

One could argue that a limitation of our study is that the dietary recall data did not provide information about whether conventional or organic foods were consumed. Organic foods have lower levels of pesticide residues. For example, in a Swiss study, 86% of the organic food samples showed no detectable glyphosate residues (Zoller et al. 2018). In consequence, exposure to pesticides including glyphosate can be anticipated to be considerably lower when mainly organic foods are consumed (Hyland et al. 2019).

In conclusion, although glyphosate is one of the most widely used pesticides worldwide, the exposure of healthy adult individuals to this pesticide in our study was very low. However, some food items, such as pulses, which are occasionally subjected to a pre-harvest treatment with glyphosate, contribute higher residue levels to the food basket, leading to higher excretion levels in adults consuming high amounts of such foods. The data presented in this study demonstrate that political decisions on permitted quantities and allowed agricultural uses/applications of pesticides might have a direct impact on individual exposure levels, despite a generally very low background of exposure.

## Electronic supplementary material

Below is the link to the electronic supplementary material.

Supplementary file1 (PDF 101 kb)

## Abbreviations

ADI: Acceptable daily intake
AMPA: Aminomethylphosphonic acid
ARfD: Acute reference dose
BVL: Federal Office of Consumer Protection and Food Safety (Germany)
cPAD: Actual chronic population adjusted dose
EFSA: European Food Safety Authority
EPA: Environmental Protection Agency
LOD: Limit of detection
LOQ: Limit of quantitation
MRLs: Maximum residue levels
NOAEL: No observed adverse effect level
