# Supplementary material for: Glyphosate and AMPA levels in human urine samples and their correlation with food consumption: results of the cross-sectional KarMeN study in Germany
Source: Arch Toxicol. 2020 Mar 30;94(5):1575–84. doi: 10.1007/s00204-020-02704-7 (PMC7261737; doi:10.1007/s00204-020-02704-7)
Supplement: Supplementary file 1 — Supplementary file1 (PDF 101 kb) [file 204_2020_2704_MOESM1_ESM.pdf]

Electronic Supplementary Material

Glyphosate and AMPA levels in human urine samples and their correlation with food consumption: Results of the cross-sectional KarMeN study in Germany

Sebastian T. Soukup[1]*, Benedikt Merz[2]*, Achim Bub[2], Ingrid Hoffmann[3], Bernhard Watzl[2], Pablo Steinberg[4], Sabine E. Kulling[1]

* These authors contributed equally to this work.

[1] Department of Safety and Quality of Fruit and Vegetables, Max Rubner-Institut, Karlsruhe, Germany.

[2] Department of Physiology and Biochemistry of Nutrition, Max Rubner-Institut, Karlsruhe, Germany.

[3] Department of Nutritional Behaviour, Max Rubner-Institut, Karlsruhe, Germany.

[4] Max Rubner-Institut, Karlsruhe, Germany.

Corresponding author: Sabine E. Kulling
Max Rubner-Institut
Haid-und-Neu-Straße 9
76131 Karlsruhe, Germany
sabine.kulling@mri.bund.de

Supplementary Material: Verification of validation parameters

Verification of validation parameters for the LC-MS/MS analyses of glyphosate and aminomethylphosphonic acid (AMPA) in urine.

Accuracy and intra-day precision (each n=6)

	32.0 µg/L urine		2.0 µg/L urine		0.2 µg/L urine	
	Accuracy [%]	Intra-day precision [%]	Accuracy [%]	Intra-day precision [%]	Accuracy [%]	Intra-day precision [%]
Glyphosate	104	2.3	102	2.6	104	10.3
AMPA	102	1.9	107	4.0	100	5.4

Inter-day precision (n=5)

The study samples as well as a quality control sample (final urine concentration of glyphosate and AMPA: 4 µg/L) were analyzed on five consecutive days. To determine the inter-day precision, the relative standard deviation of the measured concentrations of glyphosate and AMPA in the quality control samples was calculated.

	Inter-day precision [%]
Glyphosate	3.9
AMPA	6.8

Matrix effect (each n=6)

The matrix effect of a mixture of two analyte-free-urines was investigated. A value of 100% means that there is no effect of the matrix on the analyte signal. Values above 100% indicate an enhancement of the signal and values below 100% a suppression. SD, standard deviation.

	Matrix effect (Mean ± SD) [%]		
	32.0 µg/L urine	2.0 µg/L urine	0.2 µg/L urine
Glyphosate	142 ± 22	163 ± 20	157 ± 15
Glyphosate-$^{13}C_2$,^{15}N	137 ± 21	162 ± 22	157 ± 14
AMPA	146 ± 27	166 ± 29	168 ± 23
AMPA-D_2,^{13}C,^{15}N	144 ± 27	165 ± 26	161 ± 17

Linearity

Due to the lack of linearity over the whole calibration range, two calibration curves were calculated, one for the low (6 levels) and one for the high concentration range (5 levels) with final urine concentrations ranging from 0.2 to 2.0 µg/L and from 2.0 to 32.0 µg/L, respectively.

Calibration curves were obtained by linear regression using a weighting of 1/x. The correlation coefficient of the calibration curves were >0.99 and the residues of the calibration levels were <±12%.

Limit of quantitation (LOQ) and limit of detection (LOD)

LOQ was defined as the lowest calibration level (0.2 µg/L urine), which still fits the requirements for accuracy and precision. LOD was defined as the levels with a signal-to-noise (S/N) ratio of 3.

	Limit of quantitation (LOQ) [µg/L urine]	Limit of detection (LOD) [µg/L urine]
Glyphosate	0.20	0.05
AMPA	0.20	0.09

Selectivity

In accordance with Jensen et al. (2016), glyphosate and AMPA were identified in each case by their retention times and two precursor/product ion transitions for values above the LOQ. For values between LOD and LOQ (traces), just the first precursor/product ion transition (quantifier) and the retention time were used for identification because of the lower sensitivity of the second precursor/product ion transition (qualifier) compared to the first. This was accepted because Jensen et al. (2016) conducted a full validation of the method, and no interfering peaks were detected in the chromatograms at the specific retention times when verifying the method as well as when measuring the study samples.

Jensen PK, Wujcik CE, McGuire MK, McGuire MA (2016) Validation of reliable and selective methods for direct determination of glyphosate and aminomethylphosphonic acid in milk and urine using lc-ms/ms. Journal of Environmental Science and Health Part B, Pesticides, food contaminants, and agricultural wastes 51:254-259.

Supplementary Table 1: Foods reported in the present study, aggregated in food groups for further analyses.

food group	foods included
**honey**	honey
**pulses**	dried lentils and dried peas
**mushrooms**	button mushrooms (*Agaricus bisporus*), chanterelle and porcini mushrooms
**bread**	a variety of breads and buns from rye, wheat, oat, spelt or multigrain
**beer**	pils, pale ale, dark lager and wheat beer
**soy products**	tofu, soy drinks, soy sauce, soy flour, soy yoghurt and soy-based meat alternatives

Supplementary Table 2: Consumption of selected food groups by participants classified by subgroup of urinary metabolite concentrations.

	**Subgroup 1 (n=200)**		**Subgroup 2 (n=76)**		**Subgroup 3 (n=25)**		
**Variable**	**Mean**	**SD**	**Mean**	**SD**	**Mean**	**SD**	**$p_{\text{linear trend}}$**
**honey**	2.3	7.6	5.3	14.7	3.9	10.8	0.4540
**pulses**	0.9	9.5	3.2	16.3	18.8	41	<0.0001
**mushrooms**	5.3	22.6	3.8	12.9	3.1	12.1	0.6104
**bread**	140.1	114	134.9	122	164.1	121.1	0.3329
**beer**	99.6	253.7	70.3	208.6	83.2	228.1	0.7489
**soy products**	23.5	175.3	18.4	74.1	2.2	11	0.7873

Subgroup 1: Neither glyphosate nor AMPA were detected. Subgroup 2: Traces of glyphosate and/or AMPA were detected. Subgroup 3: Glyphosate and/or AMPA levels were above the limit of quantitation.

Linear regression models were used to test a linear trend across subgroups.

Supplementary Table 3: Spearman correlation coefficients and corresponding *p*-values of adjusted associations between 24-hour urine glyphosate and AMPA excretion rates and the consumption of specific food groups, adjusted for age and sex.

food group (g/day)	Glyphosate (µg/24h)		AMPA (µg/24h)		Sum Glyphosate + AMPA (µg/24h)	
	**rho**	***p***	**rho**	***p***	**rho**	***p***
**honey**	0.12	0.2078	-0.03	0.9990	0.08	0.4901
**pulses**	**0.28**	**<0.0001**	-0.004	0.9990	**0.25**	**<0.0001**
**mushrooms**	-0.07	0.5753	**0.18**	**0.0121**	0.003	0.9990
**bread**	0.03	0.9990	0.01	0.9990	0.0001	0.9990
**beer**	-0.10	0.2951	0.01	0.9990	-0.07	0.5753
**soy products**	0.001	0.9990	0.001	0.9990	-0.02	0.9990

Significant correlations are marked in **bold**; reported *p*-values are corrected for multiple testing.

Supplementary Table 4: Glyphosate and AMPA excretion rates in 24-hour urine samples of consumers and non-consumers of selected food groups.

	Glyphosate (µg/24 h)		
**food group**	**non-consumer**	**consumer**	***p***[1]
**honey**	0.085 ± 0.174	0.108 ± 0.175	0.0998
**pulses**	0.076 ± 0.151	0.443 ± 0.325	<0.0001
**mushrooms**	0.092 ± 0.176	0.069 ± 0.158	0.3379
**bread**	0.101 ± 0.186	0.087 ± 0.172	0.6278
**beer**	0.095 ± 0.181	0.058 ± 0.124	0.1227
**soy products**	0.086 ± 0.170	0.111 ± 0.203	0.8286
	AMPA (µg/24 h)		
**food group**	**non-consumer**	**consumer**	
**honey**	0.038 ± 0.128	0.012 ± 0.048	0.3106
**pulses**	0.033 ± 0.118	0.024 ± 0.078	0.8852
**mushrooms**	0.027 ± 0.110	0.079 ± 0.157	0.0016
**bread**	0.043 ± 0.145	0.031 ± 0.111	0.9314
**beer**	0.033 ± 0.123	0.029 ± 0.081	0.5525
**soy products**	0.032 ± 0.117	0.038 ± 0.116	0.7515
	Sum glyphosate + AMPA (µg/24 h)		
**food group**	**non-consumer**	**consumer**	
**honey**	0.122 ± 0.241	0.120 ± 0.202	0.3400
**pulses**	0.109 ± 0.218	0.466 ± 0.359	<.0001
**mushrooms**	0.119 ± 0.091	0.147 ± 0.056	0.8342
**bread**	0.143 ± 0.256	0.118 ± 0.229	0.5012
**beer**	0.128 ± 0.245	0.087 ± 0.153	0.3784
**soy products**	0.118 ± 0.228	0.149 ± 0.277	0.9660

All values are given as arithmetic mean ± standard deviation.

1 Mann-Whitney-U test

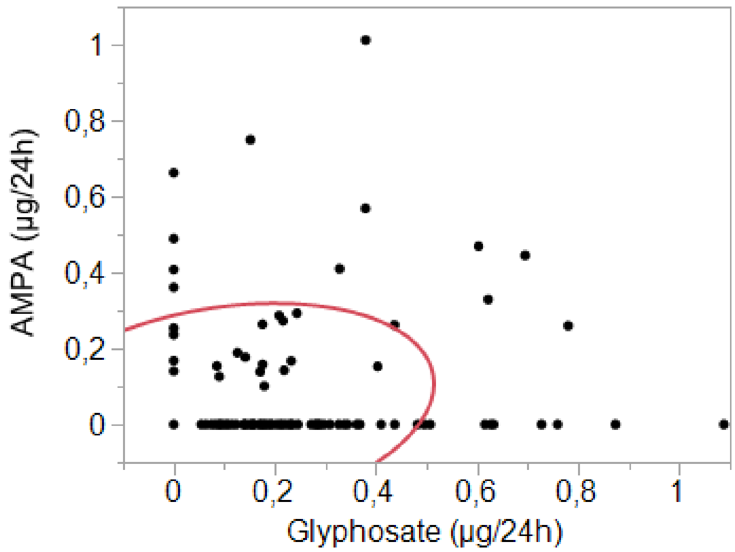

Supplementary Fig. 1: Scatter plot between the 24-hour urinary excretion of glyphosate and the 24-hour urinary excretion of AMPA (Spearman correlation rho = 0.32, $p < 0.0001$). The red circle displays the density ellipse.

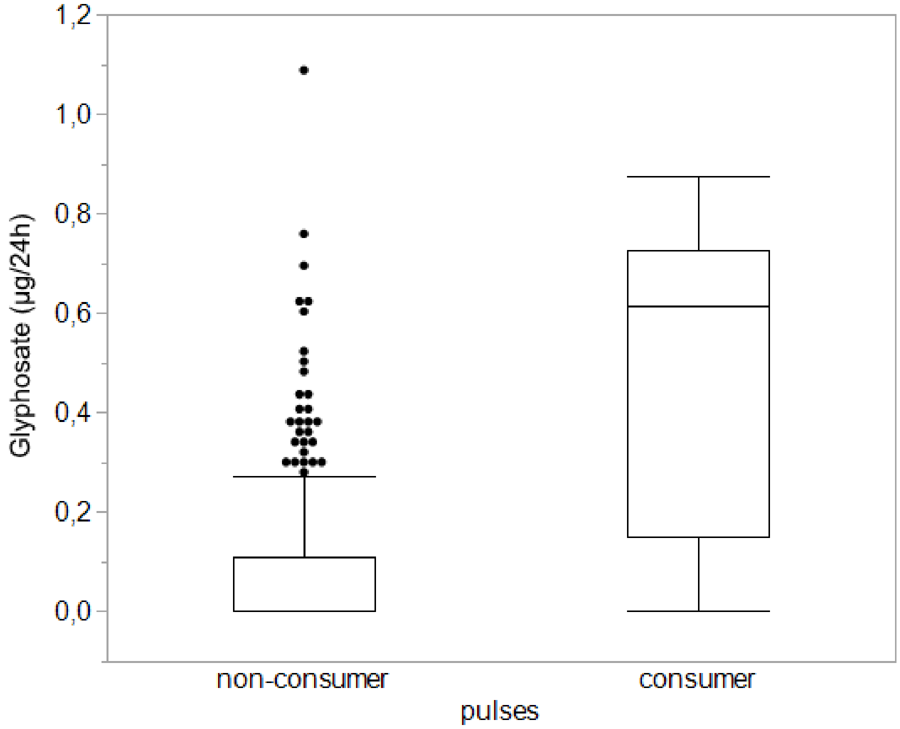

Supplementary Fig. 2: Boxplot showing differences in the 24-hour urinary levels of glyphosate for consumers and non-consumers of pulses.

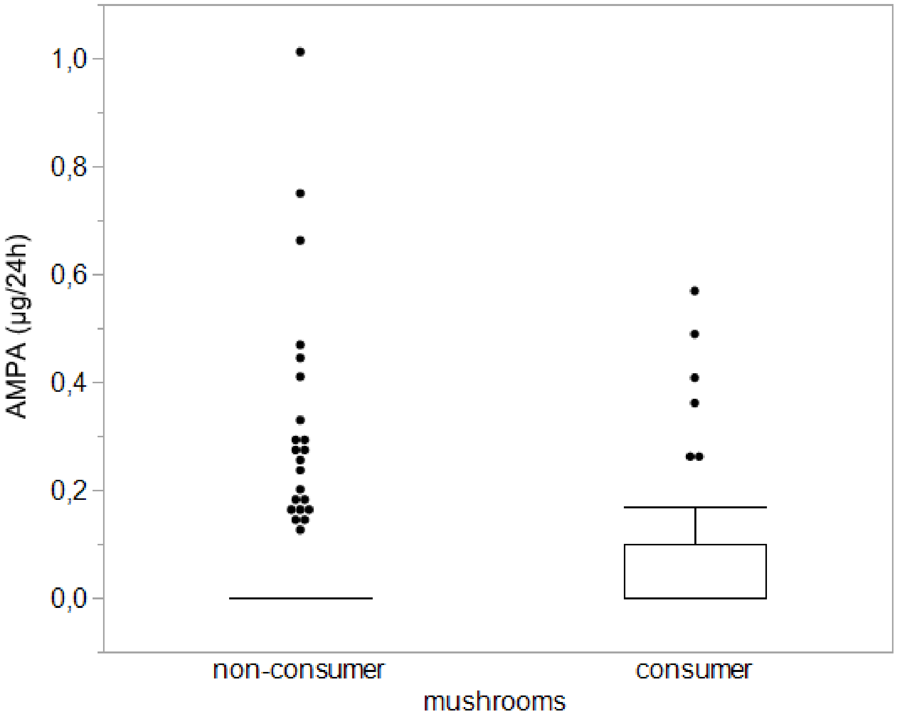

Supplementary Fig. 3: Boxplot showing differences in the 24-hour urinary levels of AMPA for consumers and non-consumers of mushrooms.

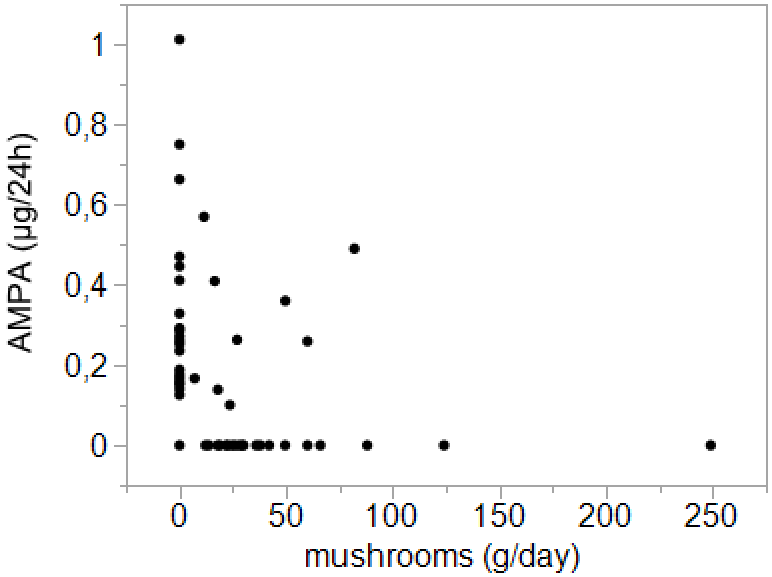

Supplementary Fig. 4: Scatter plot of the association between the consumption of mushrooms and the 24-hour urinary levels of AMPA.
